# Case report: A rare case of pure tibio-talar dislocation without malleolar clamp fracture in a young athlete: Long-term functional results and recent reviews of the literature^☆^

**DOI:** 10.1016/j.ijscr.2022.107396

**Published:** 2022-07-11

**Authors:** Walid Bouziane, Mouncef Amahtil, Mohammed Benhamou, Jawad Amghar, Soufiane Ahrram, Mohammed Sadougui, Abdessamad Lamhaoui, Jamal Karbal, Imad Bakkal, Mohammed Amine Machmachi, Omar Agoumi, Abdelkrim Daoudi

**Affiliations:** Orthopedics and Traumatology Department University Hospital Center, Mohammed VI, Oujda, Morocco; Faculty of Medicine and Pharmacy Oujda, University Mohammed first Oujda – Morocco

**Keywords:** Pure dislocation, Tibio talar, Young athlete, Case report

## Abstract

**Introduction and importance:**

Ankle dislocation without fracture is an extremely rare injury, requires high-energy trauma, up to 1995, only 73 cases were reported in the literature and 154 cases up to 2017. It is therefore a post-traumatic ankle disease growing in parallel with the increase in road-traffic accidents requiring the study of incriminated factors such as hypoplasia of the medial malleolus.

**Case presentation:**

The authors report the case of a young patient, a football player, who presented with a pure, closed dislocation of his ankle following a road-traffic accident. Management consisted of immediate reduction, followed by immobilization in plaster for 1 month,

**Clinical discussion:**

The short, medium and long term clinical and radiological results are excellent with no evidence of ankle instability or osteoarthritis.

**Conclusion:**

Ankle dislocation without associated fracture is an extremely rare injury. The posteromedial dislocation represents the most described variety in the literature. Early management, short-term immobilization, adapted and early rehabilitation seem to be associated with good long-term functional results.

## Highlights

• Ankle dislocation without fracture is an extremely rare injury, requires high-energy trauma up to 2017 , only 154 cases were reported in the literature
• The authors report the case of a young patient, a football player, who presented with a pure, closed dislocation of his ankle following a road-traffic accident
• the short, medium and long term clinical and radiological results are excellent with no evidence of ankle instability or osteoarthritis.

## 1 Introduction

Pure dislocation of the ankle, or dislocation not accompanied by fractures of the malleoli or of the posterior border of the tibia, is an extremely rare injury., requires high-energy trauma, up to 1995, only 73 cases were reported in the literature and 154 cases up to 2017. [1] D'Anca explained that this rarity was attributable to the mechanical efficiency of the mortise and the resistance of the ankle ligaments being greater than that of bone, thus causing malleolar fracture in the case of injury [2] .In the present report, we discuss a case of ankle dislocation that occurred without associated fracture in a young patient aged 21 years old that happened following an ankle traumatism during a road-traffic accident. We report also the literature review and the treatment protocol.

## 2 Case report

21 years old young, no significant pathological history, football player brought to the emergency room after being involved in a road-traffic accident with an acutely painful and obviously deformed right ankle. The diagnosis made clinically. The deformation of the foot with the deviation of the foot with respect of the leg determines an evident alteration of the normal articulation .The clinical examination had also revealed a laceration next to the lateral edge of the ankle .General, neurovascular examination showed no associated complication or abnormalities. Radiographs confirmed the clinical diagnosis of posteromedial dislocation without malleoli fracture.

**Fig. 1:**
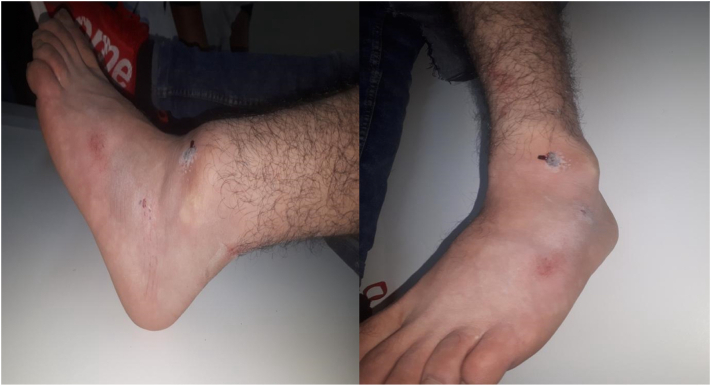
Clinically evident ankle deformity.

**Fig. 2:**
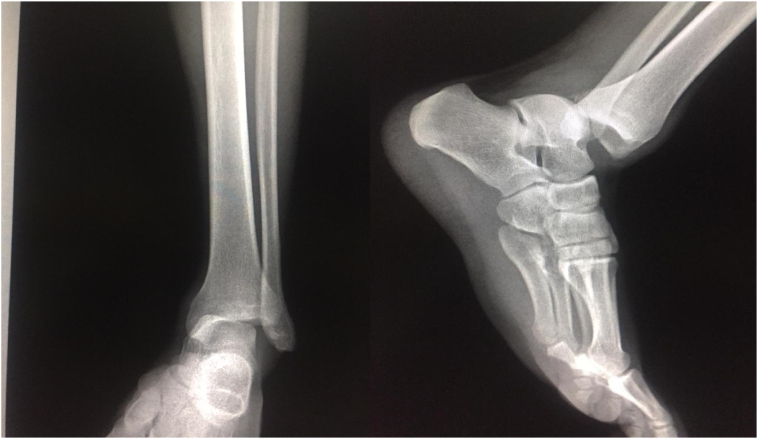
Standard front and side radiograph showing pure posteromedial tibiotal dislocation.

**Fig. 3:**
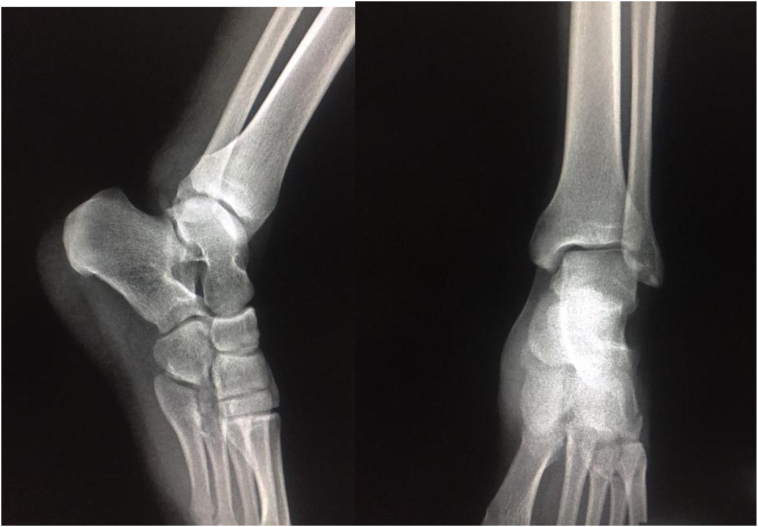
Post reduction radiograph.

**Fig. 4:**
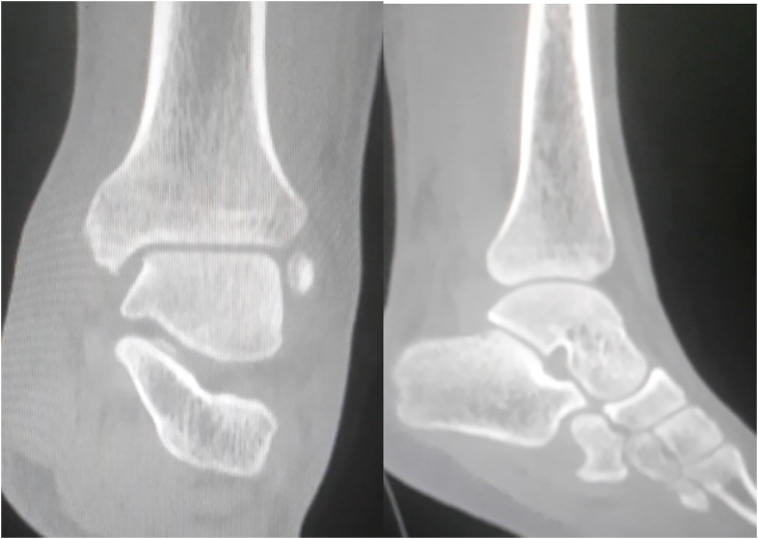
Post-reduction scan.

**Fig. 5:**
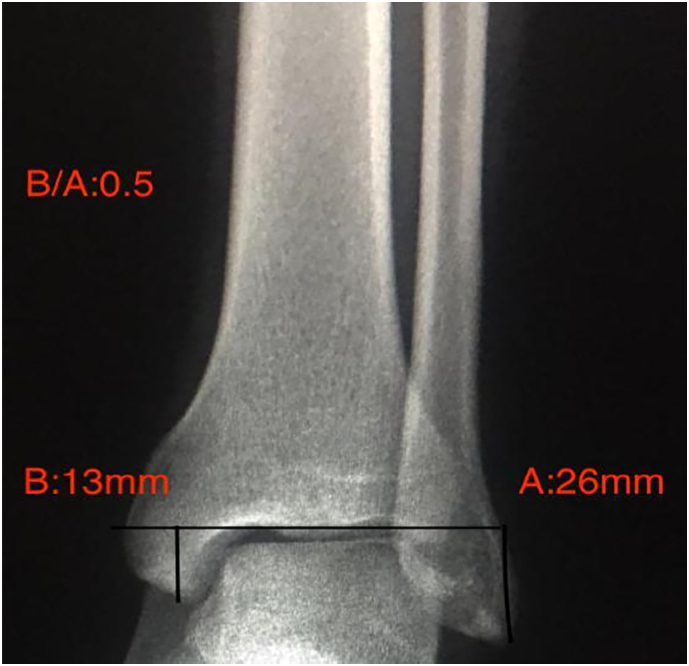
Frontal radiograph shows hypoplasia of the medial malleolus assessed with the ratio between the length of the malleolus B (medial) and A (lateral) according to the method of Elisé et al.

**Fig. 6:**
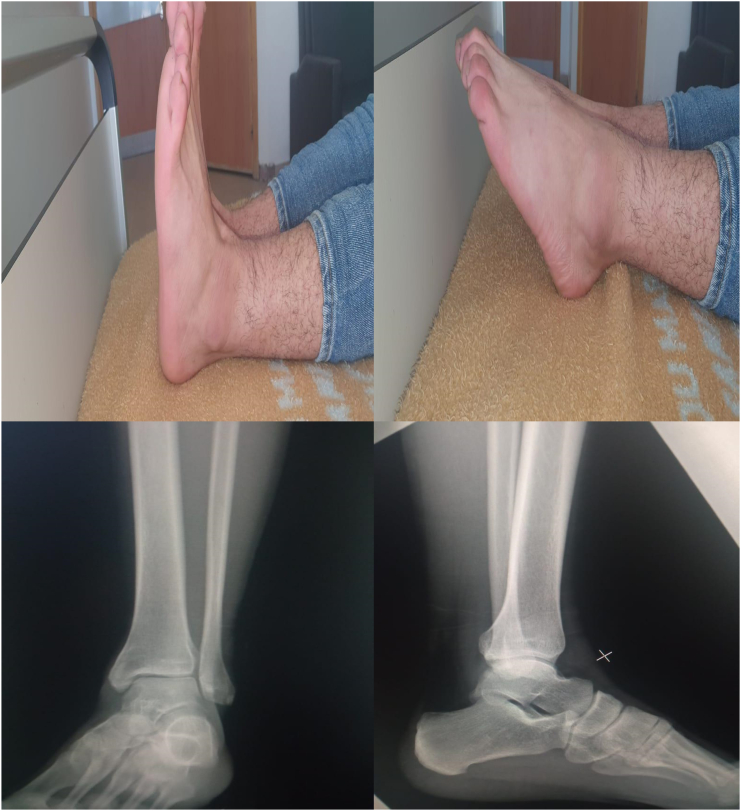
Clinical and radiological results at 3 years.

Under general anesthesia, the reduction immediately performed. by an experienced surgeon .The knee was first flexed to relax the tendon Achilles then longitudinal traction with gentle forward force applied to the heel with immediate reduction of the deformity achieved. Post reduction radiographs showed adequate reduction of the ankle joint with no talar shift or syndesmotic injury. The clinical exam confirms that there is no objective instability or ligament laxity. The evolution was marked by rapid regression of the edema of the ankle and total disappearance of the pain after 24 h. After the reduction, the joint is immobilized via the application of a short leg cast for 4 weeks followed by physical therapy and rehabilitation. The physical exam of the ankle performed after the removing of the plaster showed no laxity (Fig. 1, Fig. 2, Fig. 3, Fig. 4, Fig. 5, Fig. 6)).

## 3 Follow-up and outcomes

The patient was seen the first month, with a removal of plaster and authorization of the partial then total support after 6 weeks. The patient reported no signs of instability or painAt follow up 3-year post injury, the patient reported having no pain and made a progressive return to sports activities without any laxity or instability of the ankle. At 12 weeks, the patient achieved full range of motion (ROM) of the ankle and was able to return to normal life.

The ankle X-ray confirmed the hypoplasia of the medial malleolus evaluated with the ratio between the length of the medial (B) and lateral (A) malleolus according to the methods of Elisé et al. [1] (B/A = 0,5 current case, 0,58–0,62 normal rang).

## 4 Discussion

The ankle does not have discrete stabilization structures ventrally and dorsally. Only medially and laterally does the ankle have strong stabilization structures: the tibial and fibular malleoli and the two collateral ligamentous complexes that reinforce the thin capsule. The unstable position of the tibiotalar joint is plantar flexion because the narrow part of the talar body lies within the ankle mortise. In this position, all of the ligamentous and capsular attachments to the talus are stretched, with the exception of the posterior talofibular ligament. Sufficient force in inversion results in posteromedial displacement of the foot on the fixed tibia with tears in the anterolateral capsule attachments and in the anterior talofibular and calcaneofibular ligaments, allowing for talar ascent and tilt [1]. Likewise, an eversion force results in lateral displacement of the foot, with tearing of the medial capsule attachments and the talotibial ligament. In the case of an applied posteroanterior force on the foot in plantar flexion, the ankle is displaced anteriorly. Posterior dislocation is the result of an anteroposterior (AP) force on the foot in plantar flexion and inversion.

A review of the literature indicates that pure dislocation of the ankle is caused prevalently by road traffic accidents, while sports accidents represent the most frequent etiology of pure closed dislocations. 64 publications on this subject, with 154 cases, of which 45 are case reports and 18 case series [3], the male predominance is obvious and represents 73 % of cases with an average age estimated at 29 years with a clear male predominance (Table 1). Pure posteromedial dislocation is the most common variety with an estimated rate of 46 % (71 cases).

**Table 1:** Epidemiology of pure ankle dislocations.

Kind	Number of cases	Middle age
Male	112(73 %)	28(9–70)
Feminine	42(27 %)	30 (7–73)
Total	154(100 %)	29(7–73)

Neurovascular occurred in 30 cases (19 %) apart two cases presenting anterior tibial artery injury with limb ischemia [4]. Few patients underwent bloody reduction, capsular or lateral ankle ligament repair. Open dislocations benefited from surgical debridement with sutures of the injured anatomical structures (Table 2), immobilization was short-term in a 90° ankle cast for 4 to 6 weeks followed by early rehabilitation.

**Table 2:** Open vs. closed dislocation.

Closed vs opened	Number of cases	Male	Feminine
Closed	77(50 %)	53(69 %)	24(31 %)
Opened	76(50 %)	58(76 %)	18(24 %)
Unknown	1	1	0
Total	154 (100 %)	154	154

The most described complication in the literature is ankle stiffness [5] this may be due to long-term immobilization. The average duration of cast immobilization is 4 weeks. Studies have shown that the therapeutic strategy vis-à-vis the lateral ankle ligament after dislocation, does not overcome immobilization for 2 to 3 weeks followed by a range of rehabilitation sessions of the ankle can provide patients with results similar to surgical repair. Furthermore, studies have suggested that surgical ligament repair should be reserved for chronic ankle instabilities.

The rupture of the syndesmosis is a rare association occurring in case of superior dislocation. If it is not managed immediately, that may cause poor clinical results conditioned by the functional prognosis of ankle mortise. [1] (Table 3).

**Table 3:** Therapeutic methods.

Strategy	Simple reduction	Reduction + trimming	Ligament repair	Others	Total
Opened	4(5 %)	32(42 %)	37(48 %)	5(5 %)	77(100 %)
Closed	67(88 %)	0(0 %)	4(5 %)	5(7 %)	76(100 %)
Total	71(46 %)	32(21 %)	41(27 %)	10(6 %)	154(100 %)

Medial malleolar hypoplasia is the most incriminated risk factor in pure ankle dislocations. In fact, Elise S, and Maynou [2] performed a study on 16 cases of pure ankle dislocations with calculation of the degree of coverage of the mortise of the ankle, this lack of coverage was found in 1/3 of the cases. Other Risk factors for these injuries include excessive ankle joint laxity, weakness of the peroneals, and a history of chronic ankle sprains.

Altogether, we believe that pure ankle dislocation is a very rare entity, requiring the combination of high-energy trauma with structural abnormalities of the malleolar clamp. The hypoplasia of the medial malleolus constitutes the most detailed element in the literature. Indeed we found it in our case but this element has no impact on the prognosis of the ankle. Indeed, we insist on the surgical reduction before the six hours by an experienced surgeon and in the operating room. Immobilization for more than 4 weeks has no beneficial effect on the ankle. Immobilization for 1 month seems logical to us and largely sufficient to begin a fairly early rehabilitation. Walking is authorized as soon as the cast is removed.

## 5 Conclusion

Ankle dislocation without associated fracture is an extremely rare injury that requires high-energy traumas usually caused by an axial load through a foot in plantar flexion. The posteromedial dislocation represents the most described variety in the literature. Known risk factors for pure dislocation include hyperlaxity, medial malleolar hypoplasia, history of recurrent ankle sprains and the weakness of the peroneals. Early management, short-term immobilization, adapted and early rehabilitation seem to be associated with good long-term functional results and avoids stiffness, which remains the most common complication.

## Sources of funding

None.

## Ethical approval

None.

## Consent

Written informed consent was obtained from the patient for publication of this case report and accompanying images. A copy of the written consent is available for review by the Editor-in-Chief of this journal on request.

## Guarantor

Walid BOUZIANE.

## Research registration

Not applicable.

## Provenance and peer review

Not commissioned, externally peer-reviewed.

## CRediT authorship contribution statement

All authors contributed to the conduct of this research and read and approved the final version of t manuscript.

## Declaration of competing interest

The authors declare that there is no conflict of interest regarding the publication of this paper.

the authors have no financial, consultative, institutional, and other relationships that might lead to bias or conflict of interest.
